# Supplementary material for: ACPT gene is inactivated in mammalian lineages that lack enamel or teeth
Source: PeerJ. 2021 Jan 22;9:e10219. doi: 10.7717/peerj.10219 (PMC7831365; doi:10.7717/peerj.10219)
Supplement: Supplemental Information 2 [file peerj-09-10219-s002.pdf]

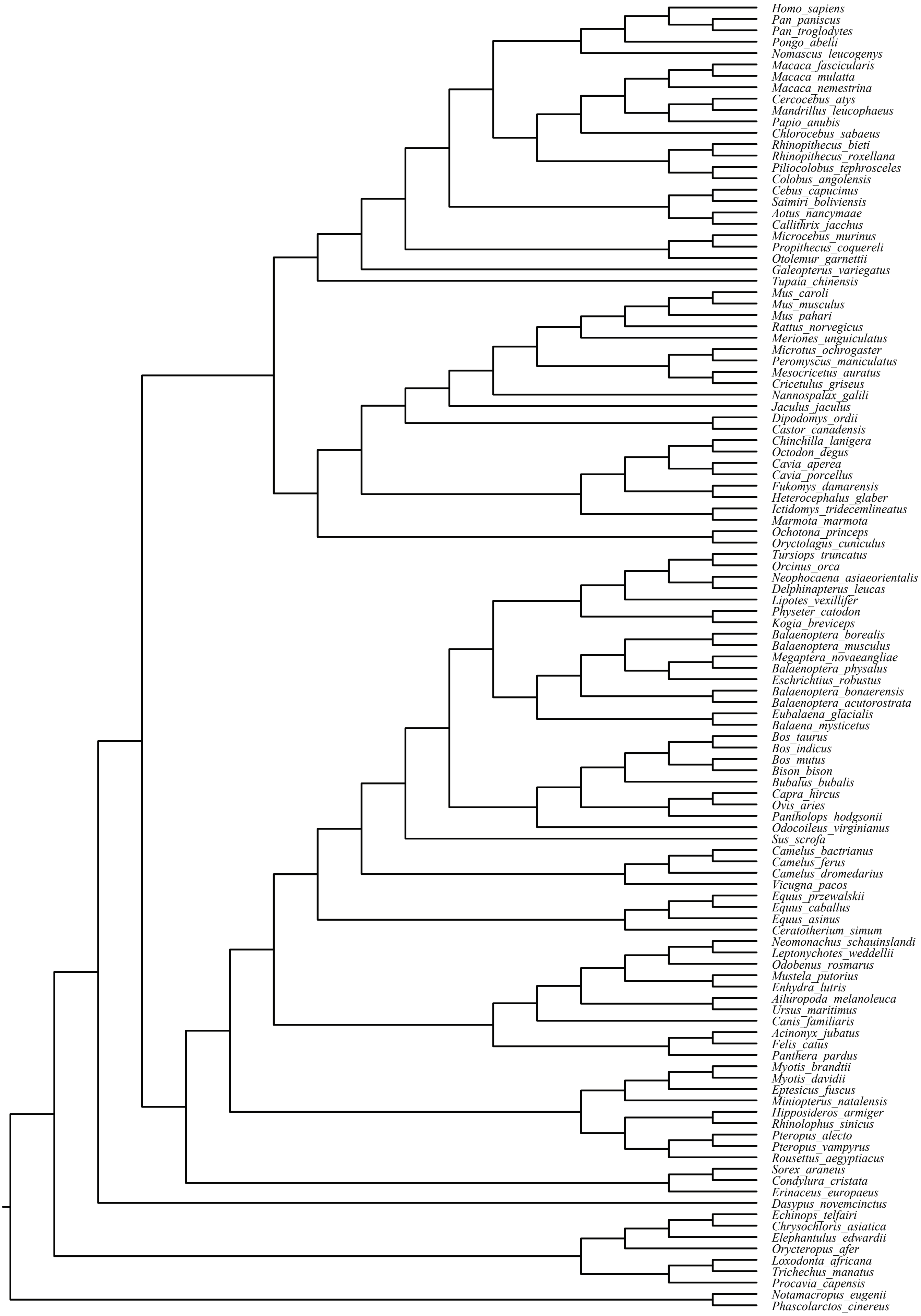

Figure S1 The tree topology of ACPT used to conduct the selective pressure analysis in PAML. The phylogeny is based on OrthoMaM and some previous researches (Celine et al., 2019; Waddell et al., 2001; Sergey et al., 2007; Zhou et al., 2012; Gatesy et al., 2013; Kuntner et al., 2011; OrthoMaM)

a.

H. sap ATGGCCGGCCTGGGGTTTTGGGGCCACCCTGCTGGACCTCTCCTGCTGCTGCTGCTGCTGGTGCTGCCACCCCGGGCCCTGCCAGAAGGA
C. hof --CCCAGGCCCTGGCGGCAGGA

H. sap CCCCTGGTGTTCGTGGCTCTGGTATTCCGCCATGGCGACCGGGCCCCGCTGGCCTCCTACCCCATGGACCCACACAAGGAGGTGGCCTCC
C. hof CCCCTGG-GTTCAGGGCTCTGG--

H. sap ACCCTGTGGCCACGAGGCCTGGGCCAGCTGACCACGGAGGGGGTCCGCCAGCAGCTGGAGCTGGGCCGCTTCCTGAGGAGCCGCTACGAG
C. hof --

H. sap GCCTTCCTGAGTCCGGAGTACCGGCGGGAGGAGGTGTACATCCGCAGCACGGACTTTGACCGCACGCTGGAGAGTGCCCAGGCCAACCTT
C. hof --

H. sap GCCGGGCTGTTTCCCGAGGCTGCTCCAGGGAGCCCCGAGGCCCGCTGG-AGGCCGATCCCGGTGCACACGGTGCCCGTGGCTGAGGATAA
C. hof -------------CCCGCGGCTGC-CCAGGGAGCCCCAGGGCCGCTGGCCGGCCCTTCCCGG---------------------------A

H. sap GCTGCTGAGGTTCCCCATGCGCAGCTGTCCCCGATACCACGAGCTGCTGCGGGAGGCCACCGAGGCCGCCGAGTACCAGGAGGCCCTGGA
C. hof GCTGCCCAGGCTCCCCATGTGCCACTGTCCCCACTGCCGGGAGCTGCTGCAGGGCTCCCCAGAGGTGGCCACGCACCAGGCAGCTCTGGA

H. sap GGGCTGGACGG---GCTTCCTGAGT-------CGCCTGGAGAACTTCACGGGACTGTCGCTGGTTGGAGAGCCACTGCGCAGGGCATGGA
C. hof GGGCTGGATGGGGAGCGGGCTGGGTGTGGTGGAGCAGGGAGCGCTTCATGGGGCTCTCACTGGTCGGGAAGCCACTCAGCAGCGTGTGGA

H. sap AGGTTCTGGACACCCTCATGTGCCAGCAAGCCCACGGTCTTCCACTACCAGCCTGGGCCTCCCCAGATGTCCTGCGGACTCTTGCCCAGA
C. hof AAGTTCTGGACACACT------CCAGCAGGCCCAGGGTCTCGCCCTCCCATCCTGGG----------TGTCCTGC--ATCCTCACCCAGA

H. sap TCTCGGCTTTGGATATTGGAGCCCACGTGGGCCCACCCCGGGCAGCAGAGAAGGCCCAGCTGACAGGGGGGATCCTGCTGAATGCTATCC
C. hof CCTCGGCTTTGGA---

H. sap TTGCAAACTTCTCCCGGGTCCAGCGCCTGGGGCTGCCCCTCAAGATGGTCATGTACTCAGCTCATGACAGCACCCTGCTGGCCCTCCAGG
C. hof --

H. sap GGGCCCTGGGCCTCTATGATGGACACACCCCGCCATATGCTGCCTGCCTCGGCTTTGAGTTCCGGAAGCACCTGGGGAATCCCGCCAAAG
C. hof --

H. sap ATGGAGGGAATGTCACCGTCTCCCTCTTCTACCGCAATGACTCCGCCCACCTGCCCCTGCCTCTCAGCCTCCCCGGGTGCCCGGCCCCCT
C. hof --

H. sap GTCCACTAGGCCGCTTCTACCAGCTGACTGCCCCGGCCCGGCCTCCCGCCCATGGGGTCTCCTGCCATGGCCCCTATGAGGCTGCCATCC
C. hof --

H. sap CCCCAGCTCCAGTGGTGCCCCTGCTGGCCGGAGCTGTAGCTGTGCTGGTGGCACTCAGCTTGGGGCTGGGCCTGCTGGCCTGGAGACCAG
C. hof --

H. sap GGTGCCTGCGGGCCTTGGGGGGCCCCGTGTGA
C. hof --------------------------------

b.

```
H. sap ATGGCCGGCCTGGGGTTTTGGGGCCACCCTGCTGGACCTCTCCTGCTGCTGCTGCTGCTGGTGCTGCCACCCCGGGCCCTGCCAGAAGGA
M. jav ---------------------------------------------------------------------------GCCCTGACGGAAGG-
M. pen ------------------------------------------------------------------------------------------
P. tri ------------------------------------------------------------------------------------GGAGG-

H. sap CCCCTGGTGTTCGTGGCTCTGGTATTCCGCCATGGCGACCGGGCCCCGCTGGCCTCCTACCCCATGGACCCACACAAGGAGGTGGCCTCC
M. jav CCCCTGGTGTTCATGGCTGTGGT-------------------------------------------------------------------
M. pen ------------------------------------------------------------------------------------------
P. tri ------------GTGTCCGC----------------------------------------------------------------------

H. sap ACCCTGTGGCCACGAGGCCTGGGCCAGCTGACCACGGAGGGGGTCCGCCAGCAGCTGGAGCTGGGCCGCTTCCTGAGGA----GCCGCTA
M. jav ------------------------------------------------------CTGCAGCTGAACAGCTTCCTGAGGA----GCCCCTC
M. pen ------------------------------------------------------CTTGGGTTTCAGGGCCTCCCCTGGACGGCGCCTCTT
P. tri ------------------------------------------------------CCGCAGCTGGACAGCTTCCTGAGGA----GCCCCTC

H. sap CGAGGCCTTCCTGAGTCCGGAGTACCGGCGGGAGGAGGTGTACATCCGCAGCACGGACTTTGACCGCACGCTGGAGAGTGCCCAGGCCAA
M. jav CCAGACCTCTCTGAGCCTGGAGTACCAGAGGGAGGAGGTGCATGTTCGCAGCC--GACTTTGGCCGGATGCTGGAGGGCGCTCAGTCCGA
M. pen CCTG---TTGCTGCTCC---AGCCCTGACGGAA---------------------------------------------------------
P. tri CCAGACCTTTTTGAGCCGGGAGTACCAGAGGGAGGAGGT---------------------------------------------------

H. sap CCTTGCCGGGCTGTTTCCCGAGGCTGCTCCAGGGAGCCCCGAGGCCCGCTGGAGGCCGATCCCGGTGCACACGGTGCCCGTGGCTGAGGA
M. jav CCT---------------------------------------------------------------------------------------
M. pen ------------------------------------------------------------------------------------------
P. tri ------------------------------------------------------------------------------------------
```

```
H. sap TAAGCTGCTGAGGTTCCCCA---TGCGCAGCTGTCCCCGATACCACGAGCTGCTGCGGGAGGCCACCGAGGCCGCCGAGTACCAGGAGGC
M. jav --GGCTAGTCTGGTTCCC-------CGAGGCCGC------TACCCCCAGCTGCTGAGGGAGGCCACCGAGGCTACGGAGTACAAGGCGGG
M. pen ------------GCTCCCTGGTGTTCGTGGCTGT----GGTACCCCCAGCTGCTGAGGGAGGCCACCGAGGCTACGGAGTACAAGGCGGG
P. tri --AGCTGCTGAGGCTCCCCA---CGCGTGGCGGTCCTCGATACCGTGAGCTGCTGAGGGAGGCCACCGAGGCTACGGAGTACAAGGGGGA

H. sap CCTGGAGGGCTGGACGGGCTTCCTGAGTCGCCTGGAGAACTTCACGGGACTGTCGCTGGTTGGAGAGCCACTGCGCAGGGCATGGAAGGT
M. jav CCTGAAGGCCTGGACG--------------------GAATTTTACGGGCCCGGCGCTGGTCTGGGAGCCGCTCCGCAGAGCGGGGAAGGT
M. pen CCTGAAGGGCTGGAGGGACTTCCCGACTTGGGGGGAGAACTTCACGAGCCCGTCGCTGGTCTGGGAGCCGCTCCGCAGGGCGGGGAAGGT
P. tri CCTGAAGGGCTGGACGGATTTCCCGACGCGGCTGGAGAACTTCACGAGCCTGTTGCTGGTCTGGGAGCCGCTCCGCAAGGCGTGGGAGGT

H. sap TCTGGACACCCTCATGTGCCAGCAAGCCCACGGTCTTCCACTACCAGCCTGGGCCTCCCCAGATGTCCTGCGGACTCTTGCCCAGATCTC
M. jav TCTGGACACCCTCATGCGCCA-----------GTCCTCCCCTCCCATCCTGGCCTCCCCCAAACGCCCTACAGACGCTAGCCCAGATCTC
M. pen TCTGGACACCCTGATGCGCCA-----------GTCCTCCCCTCCCATCCTGGGCTCCCCCAAATGCCCTACAGACACTAGCCTGGATCTC
P. tri TCTGGACACCCTGATGCGCCAGCAGGCC----GTCTTCCCCTCCCATCCTGGGCTCCCCCGAATGCCTTACGGACACTAGCCCAGATCTC

H. sap GGCTTTGGATATTGGAGCCCACGTGGGCCCACCCCGGGCAGCAGAGAAGGCCCAGCTGACAGGGGGGATCCTGCTGAATGCTATCCTTGC
M. jav AGCTTTGGATATTAAGGCTCATGTGGGCCCATCCCGGGCAGCAG---AGGCCCAGCCG--AGGGGG---CCTGCTGGATGCCATCCTTCC
M. pen AGCTTTGGATATTAAGGCTCATGTGGGCCCACCCCGGGCAGCAG---AGGCCCAGCTG--AGGGGGGATCCTGCTGGATACCA-CCTTCC
P. tri AGCTTTGGATATTAAGGCTCATGTGGGCCCACCCGGGGTAGCAAAGGAGGCCCAGCTGAGTGTGGG------------------------

H. sap AAACTTCTCCCGGGTCC-AGCGCCTGGGGCTGCCCCTCAAGATGGTCATGTACTCAGCTCATGACAGCACCCTGCTGGCCCTCCAGGGGG
M. jav CAACTTCT--CAAGTCT-AGCACCTGGGGCTGCCCCTCAAGATGGTCACCTACTCAGCTCATGACAGCA--CTACCAGCCCGCCTGGGGG
M. pen CAACTTCT--CAAGTCTAAGCACCCGGGGCTGCCCCTCAAGATGGTCACGTACTCAGCTCATGACAGCA--CTACCGGCCCGCCTGGGGG
P. tri ------------GGTCT-AGCACCTGGGGCTGCCCCTCAAGATGGTCATGTACTCAGC-CATGACAGCACACTGCCAGCCCGCCTGGGAG

H. sap CCCTGGGCCTCTATGATGGACACACCCCGCCATATGCTGCCTGCCTCGGCTTTGAGTTCCGGAAGCACCTGGGGAATCCCGCCAAAGATG
```

M. jav TCC-GGGTCTCTATGATGGGCACACCCCACCATATGCCACCTGCCTCGGC--
M. pen CCC-GGGTCTCTATGATGGGCACACCCCACCATAGGCCACCTGCCTTGGC--
P. tri CCT-GGGTCTCTGTGATGGGCACA-CCCGCCGTACGCCGCCTGCCTCGGC--

H. sap GAGGGAATGTCACCGTCTCCCTCTTCTACCGCAATGACTCCGCCCACCTGCCCCTGCCTCTCAGCCTCCCCGGGTGCCCGGCCCCCTGTC
M. jav ---GGAATGTCACAATCTCCCTCTTCTACTGCAATGACTGCACCGGCCTGCCTCTGCCCCTCAGCCTCCCTGGGTGGCCAGACCCCTGCC
M. pen ---GGAATGTCACCATCTCCCTCTTCTACTGCAATGACTGCACCGGCCTGCCTCTGCCCCTCAGCCTCCCTGGGAGGCCAGACCCCTGCC
P. tri ---GGAATGTCACAATCTCCCTCTTCTACTGCAATGGCTGCACTGGCCTGCCTCTGCCCCTCAGCCTCCGTGAGTGTCGAGACCCCTGCC

H. sap CACTAGGCCGCTTCTACCAGCTGACTGCCCCGGCCCGGCCTCCCGCCCATGGGGTCTCCTGCCATGGCCCCTATGAGGCTGCCATCCCCC
M. jav CACTAGGCCACTTCTGCCGGCTGACTGCCCT-GCCCGGTCTCCAGCTCGTGGGGTGCCCTGCTACGACTCCCATGAGTTTGCAATCCCCC
M. pen CACTAGGCCACTTCCGCCGGCTGACTGCCCT-GCCCGCCCTCCAGCTCGTGGGGTCCCCTGCCATGACTCCCATGAGTTTGCCATCCCTG
P. tri CACTAGGCCACTTCTGCCAGCTGACTGCCCTGGCCTGGCCTCCAGCTCGTGGAGTCCCCTGCCACGGCTCCCATGAGCTTGCCA------

H. sap CAGCTCCAGTGGTGCCCCTGCTGGC-CGGAGCTGTAGCTGTGCTGGTGGCACTCAGCTTGGGGCTGGGCCTGCTGGCCTGGAGACCAGGG
M. jav CAGCCACCGTCCTGCCCCCGCTGGCTTGGGGCTGCGGGTATGCCTGCAGCGCTCAGTGTGGGGCTTA------CGAACTGGAGACCTGGC
M. pen CAGGT---
P. tri --

H. sap TGCCTGCGGGCCTTGGGGGGCCCCGTGTGA
M. jav TGCCTGCCGGCCTGGGGAGGGCCTATGTGA
M. pen ---------------GGTGGCCCTGTCTGG
P. tri ------------------------------

Figure S2 a. *ACPT* sequence comparison between human and sloth;

b. *ACPT* sequence comparison among human and 3 pangolins

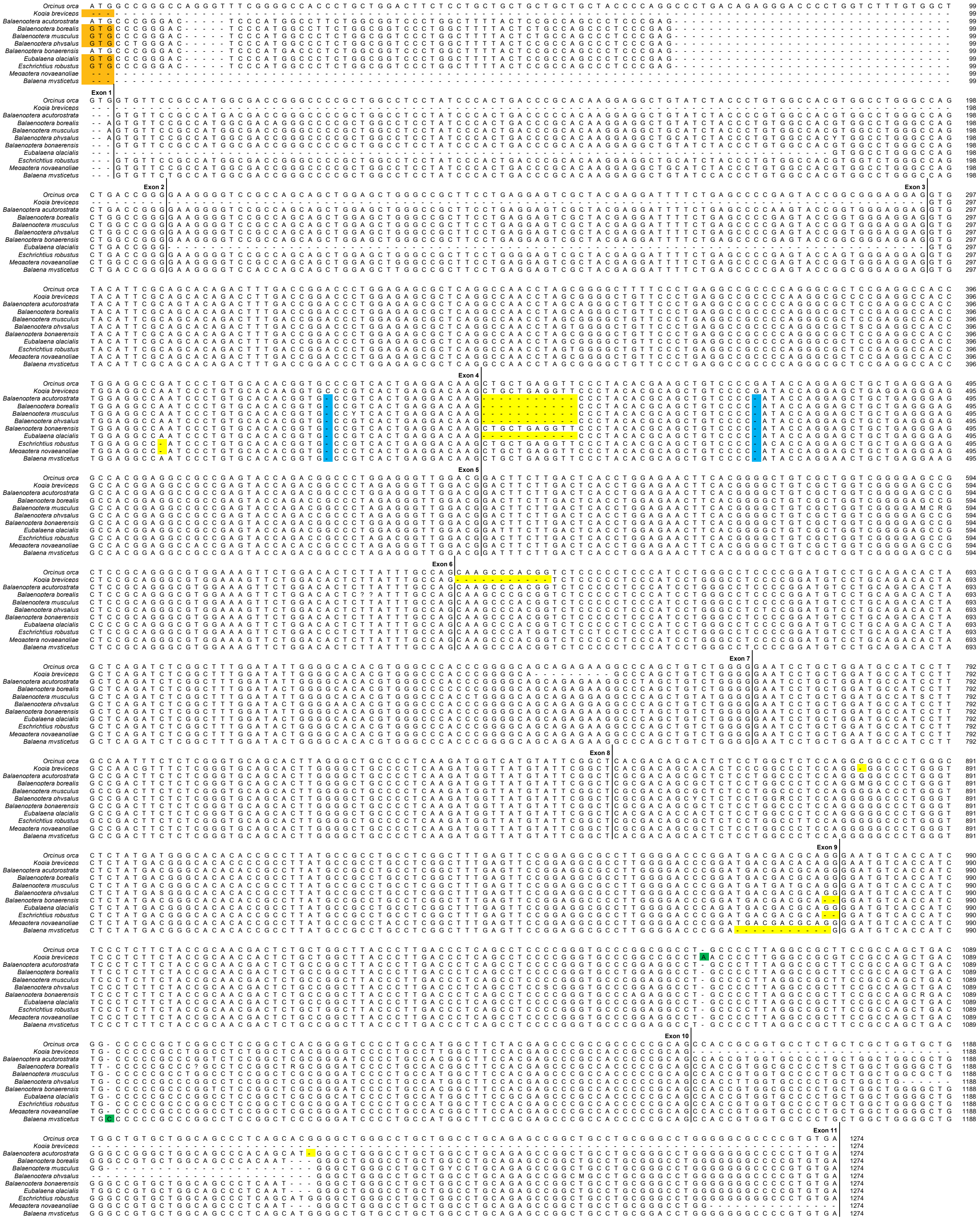

Figure S3 The detailed information about inactivated mutation of ACPT among relative cetaceans. Orange represents initiation codon mutation, yellow represents deletion, green represents insertion, and blue represents two common deletion sites among all baleen whales.

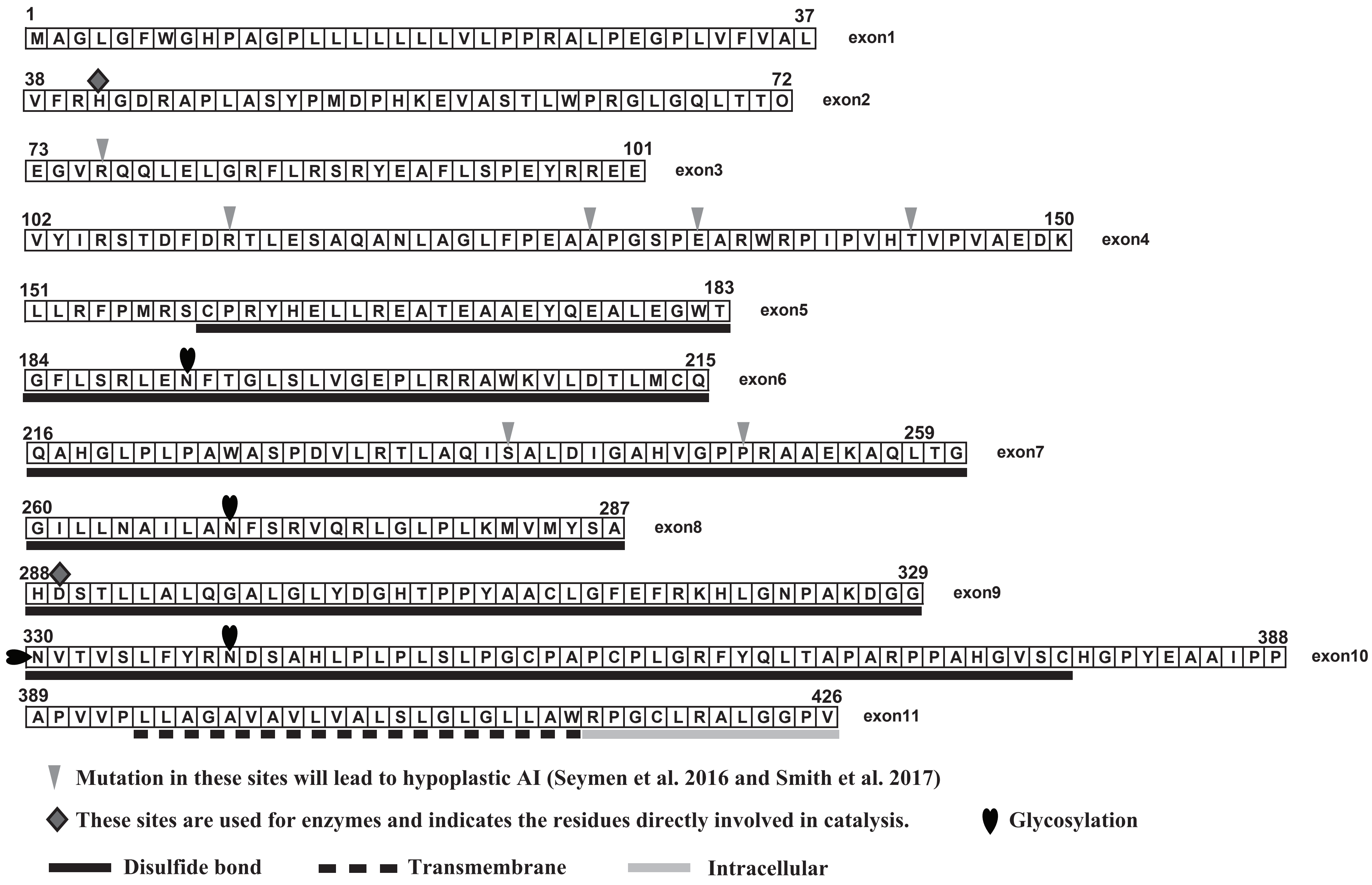

Figure S4 The information of mutation sites about amelogenesis imperfecta in ACPT protein sequence.

References

Celine S, Khalid B, Jimmy L, Rémy D, Frédéric D, Emmanuel JPD, Vincent R. 2019. OrthoMaM v10: Scaling-up orthologous coding sequence and exon alignments with more than one hundred mammalian genomes. *Molecular Biology and Evolution* **36**(4):861-862.

Gatesy J, Geisler JH, Chang J, Buell C, Berta A, Meredith RW, Springer MS, Mcgowen MR. 2013. A phylogenetic blueprint for a modern whale. Molecular *Phylogenetics and Evolution* **66**(2):479-506.

Kuntner M, May-Collado LJ, Agnarsson I. 2011. Phylogeny and conservation priorities of afrotherian mammals (Afrotheria, Mammalia). *Zoologica Scripta* **40**(1):1-15.

OrthoMaM: http://orthomam2.mbb.univ-montp2.fr/OrthoMaM_v10b10/.

Sergey N, Montoya-Burgos JI, Margulies EH, Jacques R, Bruno N, Antonarakis SE. 2007. Early history of mammals is elucidated with the ENCODE multiple species sequencing data. *PLoS Genetics* **3**(1):e2.

Seymen F, Kim YJ, Lee YJ, Kang J, Kim TH, Choi H, Koruyucu M, Kasimoglu Y, Tuna EB, Gencay K. 2016. Recessive mutations in ACPT, encoding testicular acid phosphatase, cause hypoplastic amelogenesis imperfecta. *American Journal of Human Genetics* **99**:1199-1205.

Smith CE, Whitehouse LL, Poulter JA, Brookes SJ, Day PF, Soldani F, Kirkham J, Inglehearn CF, Mighell AJ. 2017. Defects in the acid phosphatase ACPT cause recessive hypoplastic amelogenesis imperfecta. *European Journal of Human Genetics* **25**(8):1015-1019.

Waddell PJ, Kishino H, Ota R. 2001. A Phylogenetic Foundation for Comparative Mammalian Genomics. *Genome Informatics* **12**:141-154.

Zhou XM, Xu SX, Xu JX, Chen BY, Zhou KY, Yang G. 2012. Phylogenomic analysis resolves the interordinal relationships and rapid diversification of the laurasiatherian mammals. *Systematic Biology* **61**(1):150-164.
